# Inulin with different degrees of polymerization as a functional ingredient: Evaluation of flour, dough, and steamed bread characteristics during freezing

**DOI:** 10.1016/j.fochx.2024.101431

**Published:** 2024-05-03

**Authors:** Qing Yang, Jinying Guo, Fan Zhang, Fen Zhao, Gege Zhang

**Affiliations:** College of Food and Bioengineering, Henan University of Science and Technology, Luoyang 471023, Henan Province, PR China

**Keywords:** Inulin, Freezing, Dough, Steamed bread

## Abstract

In the study, the effects of short-chain inulin (OP), natural inulin (OH), and long-chain inulin (OHP) at substitution levels of 3%, 6%, and 9%, as well as freezing of 0, 15, and 30 days, on the farinograph and extensograph characteristics of flour, the rheological properties, water distribution, and microstructure of dough, as well as the quality of the final steamed bread, were investigated. The findings revealed that inulin led to a reduction in the water absorption of the dough while increasing its stable time. Furthermore, inulin delayed the alteration of freezable water within the frozen dough. Notably, the addition of inulin resulted in a more cohesive and evenly arranged network structure within the frozen dough. Steamed bread supplemented with 6% OP, 6% OH, and 3% OHP consistently dislayed a higher specific volume and spread ratio. These findings offer valuable insights into the utilization of inulin in frozen wheat foods.

## Highlights

• Freezing had an adverse effect on dough properties and the quality of steamed bread.
• Inulin altered the rheological properties and water distribution of the dough during freezing.
• Adding OP, OH, and OHP reduced the WA of the dough and improved its SQN.
• 6% OP, 6% OH, and 3% OHP effectively mitigated the damage to the gluten network.
• Adding 6% OH mitigated the degradation of dough and steamed bread quality during freezing.

## 1 Introduction

Steamed bread holds a cherished place in the Chinese diet and is revered as a traditional culinary delight. However, consuming steamed bread made from refined wheat flour often leads to a deficiency in dietary fiber, potentially contributing to health concerns such as coronary heart disease, diabetes, obesity, and constipation (Zhuang et al., 2022). Furthermore, the texture, flavor, and nutritional integrity of steamed bread often deteriorate during storage. To address these challenges, frozen dough has been employed in the production of steamed bread. The method effectively preserves the quality and flavor of steamed bread while extending its shelf life, thereby offering enhanced convenience to consumers (Zhu, 2021). Despite its benefits, products produced using frozen dough are susceptible to issues such as dryness, skin collapse, discoloration, small size, and hard texture. These changes lead to sensory deterioration in the quality of steamed bread (Ban et al., 2016).

In recent years, researchers have been addressing these issues by incorporating various additives into the frozen dough, including hydrocolloids, enzymes, emulsifiers, oxidants, and polysaccharides (Lu, Guo, Fan, Wang, & Yan, 2023). Among these additives, polysaccharides have emerged as an extensive additive to protect yeast from damage, stabilize the structure of the gluten network (Zhu, Tao, Wang, & Xu, 2023), and enhance water retention capacity (Zhang et al., 2017). However, traditional sources of dietary fiber derived from grains, legumes, fruits, and vegetables typically exhibit poor water solubility, a large particle size, and a coarse texture. These characteristics can negatively impact the appearance, taste, and overall quality of the final products (Ji, Jia, Cao, Muhoza, & Zhang, 2020). Therefore, there is a need to explore new food additives capable of not only supplementing dietary fiber but also enhancing the quality of frozen dough.

Inulin is a fructan composed of D-furanofructose molecules linked by β-(2,1) glycosidic bonds (Gonzalez-Tomás, Coll-Marqués, & Costell, 2008). It can be categorized into three types based on the degree of polymerization (DP): short-chain inulin (DP ≤ 10), natural inulin (DP = 2–60), and long-chain inulin (DP ≥ 23) (Cao et al., 2021). Recognized by the U.S. Food and Drug Administration as a food and nutritional supplement, inulin offers various physiological functions, such as lowering blood glucose, reducing blood lipids, and promoting calcium absorption (Hughes, Alvarado, Swanson, & Holscher, 2022; Liu, Luo, Chen, Xu, & Liu, 2016; Mensink, Frijlink, Maarschalk, & Hinrichs, 2015; Mudannayake, Jayasena, Wimalasiri, Ranadheera, & Ajlouni, 2022). The food industry highly values the development and utilization of inulin as a functional food additive, particularly in enhancing the quality of bread, steamed bread, and noodles (López-Castejón, Bengoechea, Alguacil, & Carrera, 2021). Inulin can significantly enhance the rheological properties of dough, decrease water absorption, prolong the dough formation time, and enhance the farinograph and extensograph characteristics of the dough (Juszczak et al., 2012; Luo et al., 2017). Additionally, inulin influenced the handling properties and consumer acceptance of bread dough (Bojnanska, Tokar, & Vollmannova, 2015). Inulin with varying DP exhibits distinct functionalities. In one study, the addition of 2.5% long-chain inulin bolstered the resistance function of proteins within the frozen dough (Ke et al., 2020). In another study, 5% natural inulin maintained the secondary structure of proteins and reduced tightly bound water (Lou et al., 2023). Furthermore, the incorporation of inulin has been shown to expedite the fermentation cycle of frozen dough, which aligns with previous findings indicating the ability of inulin to enhance yeast survival (Yang et al., 2023). Based on these, we hypothesize that inulin with various DP can enhance water migration, strengthen the gluten structure, and reduce frozen dough disruption, thereby improving the characteristics of frozen dough and ultimately enhancing the quality of the end steamed bread.

So far, research efforts have predominantly centered around investigating the influence of single type of inulin on non-frozen products. However, there is a notable scarcity of studies delving into the effects of different DP of inulin on the physicochemical properties and structure of dough and the overall quality of steamed bread, especially concerning their behavior during freezing. In the study, the effects of OP, OH, and OHP at substitution levels of 3%, 6%, and 9%, as well as with freezing of 0, 15, and 30 days, on the farinograph and extensograph characteristics of flour were investigated. Additionally, the effects of inulin and freezing on the rheological properties, water distribution, and microstructure of dough, as well as the quality of the final steamed bread, were explored. Consequently, the mechanism of inulin and the freezing effect on the characteristics of dough and the quality of steamed bread were investigated. These studies provide valuable insights into the potential applications of inulin in frozen wheat-based food production.

## 2 Materials and methods

### 2.1 Material

The plain wheat flour (‘Xiang Nian Brand’, harvested in 2020) utilized in this experiment comprised 1.63% fat, 12.15% protein, 66.48% starch, 13.04% water, and 0.31% ash (dry basis, *w*/w). These values were determined using methods 922.06, 920.87, 996.11, 925.10, and 923.03, respectively, as outlined in the AOAC (2000) guidelines. Orafti®P95 inulin (OP, average DP ≤ 10, inulin content 95%) was procured from Beijing Houjia Biotechnology Co., Ltd. (Beijing, China), Orafti®HIS inulin (OH, DP 2–60, inulin content 88%) was procured from New Nutrition Beijing Technology Co., Ltd. (Beijing, China), and Orafti®HP inulin (OHP, average ≥ 23, inulin content 100%) was procured from Wuhan Bo Rui Yuan Biotechnology Co., Ltd. (Wuhan, China). Instant dry yeast was purchased from Angel Yeast Co., Ltd. (Yichang, Hubei, China). All chemicals were of analytical grade unless specified.

### 2.2 Preparation of wheat flour-inulin blends

The compound blend was prepared by partially replacing wheat flour with three types of inulin with different DP. Inulin levels of 3%, 6%, and 9% (g/g, flour based, dry basis) were used. The amount of inulin added was determined based on the results of preliminary experiments. The inulin was dried in an oven at 60 °C until it reached a constant weight. Subsequently, it was mixed with the flour at 25 °C in a kneader (HM740, Qingdao Hanshang Electric Co., Ltd., Qingdao, Shandong, China). The sample without the addition of inulin served as the control group.

### 2.3 Farinograph and extensograph

The farinograph characteristics of the dough during the formation process were tested using an *E*-farinograph (JFZD-300, Beijing Dongfu Jiuheng Instrument Technology Co., Ltd., Beijing, China) following the AACC (2010)

. The water absorption (WA), dough development time (DT), stable time (ST), softening degree (SD), and farinograph quality number (FQN) of flour-inulin blends were determined.

The extensograph parameters of the dough were performed using an extensograph (JMLD-150, Beijing Dongfu Jiuheng Instrument Technology Co., Ltd., Beijing, China). The dough prepared with an E-farinograph was divided into a 150 g piece and fermented for testing over a period ranging from 45 to 135 min. The dough extensibility (E), resistance to extension (R), ratio of resistance to extensibility (R/E), and extension energy were obtained. Farinograph and extensograph measurements were performed in three repetitions.

### 2.4 Frozen dough preparation

The control dough formula consisted of 100 g of wheat flour, 1 g of dry yeast, and an appropriate amount of distilled water. Based on the results of the farinograph test, the water added to the inulin wheat flour mixture constituted 95% of the water absorption rate. All mentioned ingredients were combined at 60 rpm/min for 3 min and then at 90 rpm/min for 4 min in a kneader (HM740, Qingdao Hanshang Electric Co., Ltd., Qingdao, Shandong, China) until the gluten network formed. After 5 min of resting, the dough was divided into 50 g portions, shaped, and then wrapped in plastic wrap. The obtained dough was swiftly frozen at a temperature of −70 °C for 1 h in a rapid freezing cabinet (DW-86 L486, Qingdao Haier Electric Appliance Co., Ltd., Qingdao, Shandong, China) and subsequently transferred to a medical cryogenic refrigerator (BCD-187L9RSZ, Hefei Royalstar Electronic Technology Co., Ltd., Hefei, Anhui, China) at −18 °C for storage of 0, 15, and 30 days, respectively.

### 2.5 Rheology

The rheological properties of dough were analyzed using a rotary rheometer (DHR-2, New Castle, DE, USA) equipped with the geometry of parallel plates (diameter 40 mm and 2 mm clearance) as previously described (Peng, Li, Ding, & Yang, 2017). The frozen dough was thawed in a tray at 25 °C for 1 h. Subsequently, a central portion of approximately 5 g was extracted and then wrapped in plastic wrap. The dough was placed between the plates and rested for 5 min to relax the residual pressure. To prevent drying during the test, a fine coating of mineral oil was applied. The dough was then measured with a scanning frequency of 0.1–100 Hz under a 0.5% strain (within the linear viscoelastic region) at 25 °C. The storage modulus (G'), loss modulus (G"), and loss tangent (tan δ = G"/G') were obtained. Each sample was measured in three replicates.

### 2.6 Low field nuclear magnetic resonance

The water distribution of dough was measured using a low field nuclear magnetic resonance (LF-NMR) (NMI20-015 V-I, Shanghai Newmai Electronic Technology Co., Ltd., Shanghai, China) by the method (Duan et al., 2024) with minor modifications. The center of the dough (about 3 g) was kneaded into strips, wrapped in plastic wrap, and then placed in an NMR cuvette (15 mm in diameter). Transverse relaxation time (T_2_) was measured under the Carr-Purcell-Meiboom-Gill sequence and calibrated with standard oil samples. The SW was 200 kHz, SF was 21 MHz, RFD was 0.08 ms, RG1 was 20.0 db, DRG1 was 3, PRG was 1, TW was 3500 ms, NS was 16, TE was 0.2 ms, and NECH was 2000. The same level was measured three times.

### 2.7 Scanning electron microscope (SEM)

The microstructure of the dough was observed using a scanning electron microscopy (JSM-5410LV, Tokyo, Japan) by the method (Guo et al., 2022). The sample was freeze-dried in a freeze-dryer (LGJ-10D, Beijing Sihuan Scientific Instrument Factory Co., Ltd., Beijing, China) for 24 h and then cut into approximately 1 cm × 1 cm × 1 cm pieces. Subsequently, the dough samples were coated with sputtered gold and viewed at magnifications of 500 × and 1500 × .

### 2.8 Preparation of steamed bread

The steamed bread was prepared as previously described (He et al., 2020) with minor modifications. The frozen dough was thawed in a fermentation chamber (YH-13C, Guangzhou Special Trading Co., Ltd., Guangzhou, China) at 30 °C and 85% humidity for 40 min. Each dough was then kneaded 60 times by hand and placed in an aluminum steamer (QVL1530, Aisda Group Limited Co., Ltd., Taizhou, Zhejiang, China) to be steamed over boiling water for 20 min. The steamed bread was taken out and cooled at 25 °C for 1 h for further analysis.

### 2.9 Colorimetry of steamed bread

The crust color of the steamed bread was determined using a colorimeter (Color i5, X rite, USA) by a method (Koksel et al., 2023). The prepared steamed bread was divided into 15 mm thick pieces, and the crust was carefully torn off and further divided into three pieces. Subsequently, the L* (0: black, 100: white), a* (−a*: greenness; +a*: redness), and b* (−b*: blueness; +b*: yellowness) of steamed bread were measured. The same treatment was performed in three replicates.

### 2.10 Specific volume and spread ratio of steamed bread

The weight (g) and the volume (mL) of steamed bread were determined by the millet replacement method. The specific volume of steamed bread was obtained by dividing the volume by the weight. Each sample was tested with three replicates.

The spread ratio of steamed bread was measured with vernier calipers by the method (Ayo-Omogie, 2021). Each sample of steamed bread was measured three times at three distinct locations. The spread ratio was calculated by using Eq. (1):

(1) S=h/d

Where, *S* is the spread ratio of steamed bread; *h* is the height (mm) of steamed bread; *d* is the diameter (mm) of steamed bread.

### 2.11 Texture of steamed bread

The textural properties of steamed bread were measured using a texture analyzer (TA-XT, Stable Micro Systems Ltd., Surrey, UK) equipped with a 1 kg load cell and an aluminum cylindrical probe (P/36D) as previously described (Zhang et al., 2024). The steamed bread was sliced into 15 mm thick sheets, and cores measuring approximately 1.5 cm × 1.5 cm × 1.5 cm were extracted for texture measurement. The pretest speed was 1.0 mm/s, the test speed was 1.0 mm/s, the posttest speed was 5.0 mm/s, the compression ratio was 60.0%, the time interval before and after two actions was 5.0 s, and the triggering force was 5.0 g. The hardness, springiness, cohesiveness, and resilience were calculated by the texture profile analysis curve. Texture measurements were performed in three replicates.

### 2.12 Statistical analysis

The data processing and graphical representations were conducted using Origin software version 8.5 (OriginLab Corp., MA, USA). Statistical analyses were performed using SPSS 19.0 (IBM Inc., Chicago, IL, USA) with a significance level of *P* < 0.05.

## 3 Results and discussion

### 3.1 Farinograph properties of dough

The farinograph characteristic is a key indicator for evaluating the properties of dough (Tao, Zhu, Nan, Jiang, & Wang, 2021). The effects of different inulin additions on the farinograph properties of dough are shown in Table S1. The water absorption (WA) value of the dough with added inulin was significantly (*P* < 0.05) lower than that of the control, likely due to the good hydrophilicity of inulin (Morris & Morris, 2012). WA displayed a declining trend with the increase in inulin substitution. When the substitution level of OP, OH, and OHP was 9%, the WA of dough decreased by 16.65%, 20.34%, and 15.08%, respectively, compared with that of the control. This is because of the formation of a barrier by inulin around the starch particles. The barrier hinders the interaction between the starch particles and water molecules, ultimately leading to a reduction in the dough's WA capacity. The development time (DT) of dough was first increased and decreased with the increase of OP and OHP substitution levels, while it first decreased and then increased with the increase of OH substitution levels. This is because inulin competes with protein or starch for water, thereby influencing the formation of the gluten network and resulting in an extended dough DT (Brennan, Kuri, & Tudorica, 2004).

Here, adding inulin significantly (*P* < 0.05) increased the stable time (ST) value of dough compared with the control group (Table S1). Specifically, when the substitution rate of OH reached 9%, the ST surged to 17.3 min, showcasing a 1.84 times enhancement over the control duration of 9.4 min. Moreover, the ST for the 3% OP and 3% OHP samples was 1.36 and 1.40 times that of the control, respectively. These findings suggest that inulin increases the dough's resistance to shear strain during the mixing process.

The softening degree (SD) represents the resistance of the dough to the shear force generated by mechanical mixing. The lower the value of the SD, the worse the quality of the gluten. The incorporation of inulin significantly (*P* < 0.05) decreased the SD value of the dough compared with that of the control. The dough added with 6% OP, 9% OH, and 3% OHP reached the lowest SD value, which decreased by 33.8%, 53.8%, and 35.4%, respectively, compared with the control. The reduction is attributed to inulin enhancing the strength of the dough, resulting in a more robust gluten network structure and improving the processing performance of the dough (Zhang, Mei, Chen, & Chen, 2017). Here, the FQN value of the dough with inulin was significantly (*P* < 0.05) higher than that of the control, indicating that all three types of inulin improve the farinograph properties of flour. Specifically, the FQN values of the dough decreased with increasing OP and OHP substitution, while their values initially decreased and then increased with increasing OH inulin substitution. These findings indicate that the value of FQN is strongly influenced by the amount of inulin substitution.

Overall, the incorporation of inulin improves the DT and ST of the dough and reduces the water absorption and SD of the dough.

### 3.2 Extensograph properties of dough

**Fig. 1:**
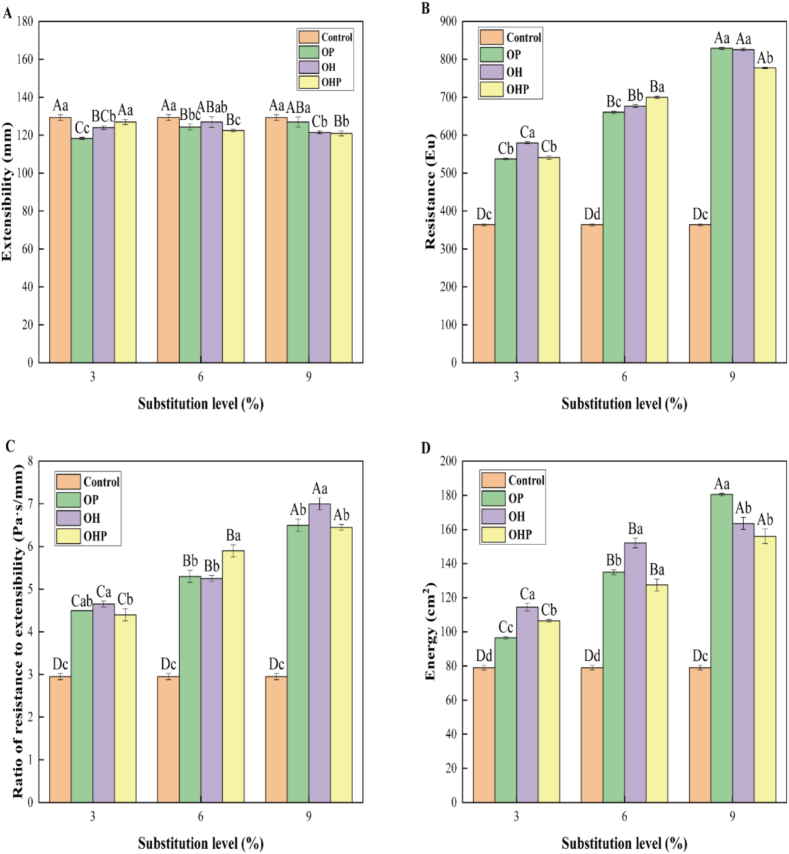
**Eextensibility, Resistance, Ratio of resistance to extensibility, and Energy of dough containing inulin after a fermentation time of 135 min**. OP, wheat flour with short-chain inulin; OH, wheat flour with natural inulin; OHP, wheat flour with long-chain inulin. Error bars represent the mean standard deviations of triplicate determinations. Different upper case letters indicate significant (*P* < 0.05) differences between different substitution amounts for the same DP. Different lower case letters indicate significant (*P* < 0.05) differences between different DP for the same amount of substitution.

The stretching curve of dough characterizes the strength and resistance to elongation conferred by glutenin, along with the ease of flow and adhesion needed for elongation as provided by glutenin (Gharaie, Azizi, Barzegar, & Aghagholizade, 2015). In a certain range, the larger the value of the dough's extensibility, ratio of resistance to extension, and extension energy, the better the tensile property of the dough. The influence of three types of inulin on the extensograph properties of dough is shown in Fig. 1. The R, the R/E, and the extension energy of the dough containing inulin significantly (*P* < 0.05) increased compared with those of the control. These findings indicate that inulin is hydrated at the appropriate temperature and pH and forms viscous and slippery macromolecular substances with gluten (Rushchitc, Shcherbakova, & El-Sohaimy, 2022).

Here, the E of dough refers to its ability to stretch without breaking, indicating its fluidity and flexibility during handling. When dough possesses good E value, it becomes more pliable and less prone to tearing or breaking during manipulation. The E value of the dough containing inulin was significantly (*P* < 0.05) lower than that of the control (Fig. 1A). This is attributed to the viscous and gel properties of the inulin itself. In one study, the E of soft dough gradually decreased with an increase in the substitution amount of inulin (Luo et al., 2018). This change could be explained by the interaction between inulin and wheat protein dough substrates, such as glutenin and gliadin (Ahmed, Thomas, & Khashawi, 2020).

The R in dough reflects its toughness. Optimal R in the dough is crucial for facilitating proper fermentation and attaining the desired volume in the final baked product. The R of the dough gradually increased with the increase in the inulin substitution level (Fig. 1B). These findings indicate that inulin improves the tensile strength and toughness of wheat gluten. The R/E indicates the relationship between the tensile strength and the ductility of the dough, and the larger extension energy means a stronger resistance to mixing, which can produce better flour baking quality. The R/E value gradually increased with the increase in the inulin substitution level (Fig. 1C), indicating that inulin enhances the dough's elongation resistance. When the fermentation time was 135 min, the extension energy value of dough with 9% OP, 9% OH, and 9% OHP substitution reached its maximum, which was 2.28, 1.92, and 1.97 times that of the control, respectively (Fig. 1D). This may be due to the involvement of inulin in the network structure of gluten, which promotes the formation of disulfide bonds and enhances the stability of the bonds formed between proteins.

In summary, the incorporation of inulin with varying DP enhances the extensograph properties of dough. Additionally, inulin promotes the formation of the gluten network structure and enhances the strength of gluten.

### 3.3 Rheological properties of dough

**Fig. 2:**
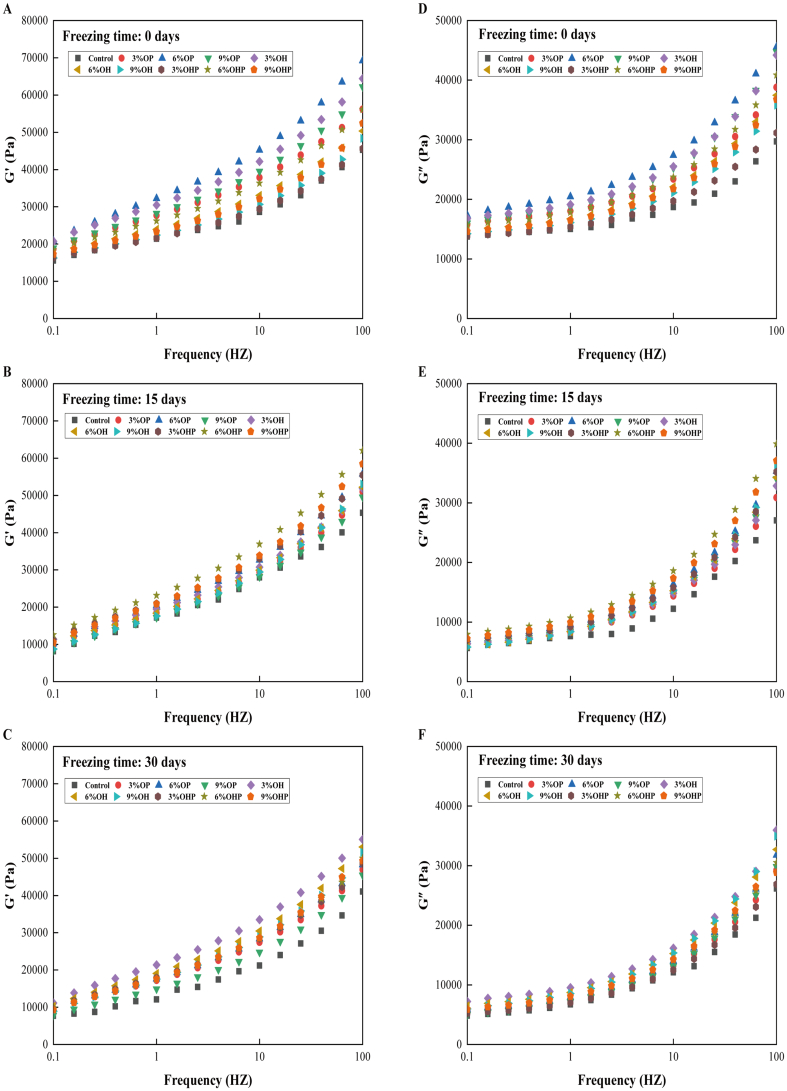
**Dynamic rheological characteristics of dough containing inulin after being frozen for 0 days (A) and (D), 15 days (B) and (E), and 30 days (C) and (F).** OP, dough with short-chain inulin; OH, dough with natural inulin; OHP, dough with long-chain inulin.

The G' reflects the material's ability to store and recover energy during deformation, highlighting its elastic behavior. Meanwhile, the G" denotes the energy dissipated or lost during deformation, depicting the material's viscous nature (Muadiad & Sirivongpaisal, 2021). Dough is a viscoelastic material, displaying characteristics of both viscous fluids and elastic solids. Fig. 2 illustrates the effects of adding short-chain, natural, and long-chain inulin on the viscoelasticity of dough frozen for 0, 15, and 30 days, respectively.

For the unfrozen dough, the G' of dough with 6% OP addition was higher than that of 6% OHP when the scanning frequency was 100 Hz (Fig. 2A), indicating that OP has a better effect on dough viscoelasticity than OHP. In one study, the G' and G" of dough with long-chain inulin were significantly higher than those of short-chain inulin at the same concentration, indicating that the enhancement of dough viscoelasticity is more pronounced with higher polymerization degrees of inulin (Liu et al., 2022). This contrasts with the findings of this experiment. This difference is attributed to the sensitivity of the dynamic modulus to water content (Peressini & Sensidoni, 2009). Here, the G' of the control presented a declining trend with increasing freezing time. One possible reason is that the gluten network of the dough is disrupted by the formation and recrystallization of ice crystals during frozen storage (Xin, Nie, Chen, Li, & Li, 2018). At the same freezing time, the G' of the dough containing inulin was higher than that of the control dough, indicating that the addition of inulin improves the elasticity of the dough and the gluten network structure. From 0 to 30 days of freezing, the G' of the control, 3% OP, 6% OH, and 3% OHP dough decreased by 50.69%, 49.41%, 41.14%, and 41.62%, respectively. These findings suggest that the incorporation of inulin impedes the development and reformation of ice crystals while enhancing the quality of the frozen dough.

Here, the G' and G" of dough increased with the increase in the substitution level of the three types of inulin, indicating that inulin promotes the cross-linking reaction of gluten, thereby improving the viscoelasticity of dough. The G" of the dough initially increased and then decreased with the increased substitution of OP and OHP, reaching its peak at the 6% substitution. Conversely, the G" value of the dough displayed a decreasing trend with the increase in OH substitution (Figs. 2D-F). These findings suggest that different types of inulin have distinct effects on the viscous properties of the dough during freezing. This understanding is crucial for optimizing these ingredients to achieve desired dough characteristics during freezing. Inulin, being hydrophilic, interacts with water molecules, starch, and proteins in the dough, leading to the formation of a complex structure within the dough matrix. Consequently, these changes lead to alterations in the rheological properties of the dough (Yue et al., 2023). At the scanning frequency (0.1–100 Hz), the value of G" was significantly lower than that of G', indicating that the dough is a solid with viscoelastic characteristics (Qin, Gao, Zeng, Liu, & Dai, 2021). The G" of dough decreased with the increase in freezing time and reached its minimum after 30 days of freezing. This is because freezing destroys the gluten structure and reduces the viscoelasticity of the dough (Muadiad & Sirivongpaisal, 2021).

### 3.4 Water distribution of dough

The moisture content and distribution of water within the dough are pivotal factors that dictate the quality of both the frozen dough and the final product (Lin et al., 2021). The formation of ice crystals is notably affected by the water's state within the dough during freezing. The size and structure of ice crystals can profoundly influence the dough's integrity and overall quality (Duan et al., 2024). The relaxation time T_2_ serves as an indicator of water fluidity in various states within the dough. A lower T_2_ value indicates improved water retention, tighter internal binding of water, and reduced water fluidity. T_21_, T_22_, and T_23_, ranging from 0.01 to 4 ms, 4 to 50 ms, and 50 to 200 ms, represent bound water, semi-bound water, and free water within the dough, respectively (Yang et al., 2021). The ratio of each peak area to the total integral area (A_21_, A_22_, and A_23_) signifies the relative percentage content of different water divisions within the dough.

The effect of inulin on the water distribution of dough is shown in Table 1. The T_21_ of frozen dough containing inulin was significantly (*P* < 0.05) lower than that of the control, indicating that inulin reduces the freedom or mobility of this particular portion of water within the dough. This reduction suggests that inulin plays a role in altering the water distribution and mobility, which can impact the dough's texture and stability. The T_23_ value of unfrozen dough containing inulin was lower than that of the control. This is because of the strong hydrophilicity and water retention properties of inulin (Majzoobi, Aghdam, Eskandari, & Farahnaky, 2019). The hydroxy group of the molecular chain diminishes water fluidity and relaxation time by engaging in proton exchange with water molecules. This impact is notably distinct for OHP, likely due to its stronger interaction with macromolecules like proteins and sugars compared with OP and OH (Liu et al., 2016). The T_23_ value of the frozen dough containing inulin was higher compared with that of the control at the same freezing time. The T_22_ of the control dough exhibited an increasing trend with the duration of freezing time. This observation is attributed to the formation of ice crystals during freezing, which disrupts the gluten network and accelerates water migration within the dough (Jiang et al., 2019).

**Table 1:** Water distribution of dough containing inulin at different freezing time.

Freezing time (day)	Inulin	Substitution level (%)	T_21_ (ms)	T_22_ (ms)	T_23_ (ms)	A_21_ (%)	A_22_ (%)	A_23_ (%)
0	Control	0	2.26 ± 0.07^a^	14.17 ± 0.00^b^	110.10 ± 0.17^a^	18.74 ± 0.33^c^	77.48 ± 0.71^a^	2.89 ± 0.24^c^
	OP	3	2.00 ± 0.00^bc^	13.55 ± 1.06^c^	97.77 ± 0.70^bc^	18.92 ± 0.31^c^	76.83 ± 0.02^d^	1.76 ± 0.45^c^
		6	2.00 ± 0.00^bc^	14.17 ± 0.00^b^	100.14 ± 0.70^c^	21.44 ± 1.43^a^	79.45 ± 0.00^b^	2.25 ± 0.86^abc^
		9	2.00 ± 0.28^bc^	14.17 ± 0.00^b^	87.77 ± 0.32^d^	20.96 ± 0.87^ab^	76.13 ± 0.02^d^	1.66 ± 0.19^c^
	OH	3	1.92 ± 0.15^bc^	14.17 ± 0.00^b^	107.34 ± 0.52^a^	18.79 ± 0.64^c^	80.17 ± 0.06^b^	1.79 ± 0.48^abc^
		6	2.00 ± 0.00^bc^	14.17 ± 0.00^b^	86.97 ± 0.00^de^	19.58 ± 0.33^bc^	81.32 ± 0.19^a^	2.75 ± 0.26^ab^
		9	1.83 ± 0.07^c^	14.78 ± 014^a^	95.85 ± 0.63^bc^	19.91 ± 0.48^abc^	78.43 ± 0.07^c^	2.26 ± 0.84^abc^
	OHP	3	2.10 ± 0.17^ab^	14.17 ± 0.00^b^	81.31 ± 8.01^e^	20.45 ± 0.00^abc^	78.23 ± 0.01^c^	2.50 ± 0.26^abc^
		6	2.00 ± 0.00^bc^	14.17 ± 0.00^b^	86.51 ± 1.36^de^	20.68 ± 1.09^ab^	77.09 ± 0.11^d^	2.64 ± 0.51^abc^
		9	1.92 ± 0.15^bc^	14.17 ± 0.00^b^	91.81 ± 2.12^cd^	19.47 ± 0.05^bc^	76.16 ± 1.07^d^	2.91 ± 0.40^a^
15	Control	0	2.15 ± 0.00^a^	14.32 ± 0.00^a^	120.22 ± 1.05^g^	12.60 ± 0.14^d^	86.57 ± 0.00^a^	1.58 ± 0.00^a^
	OP	3	1.82 ± 0.07^e^	14.23 ± 1.50^ab^	156.22 ± 0.32^b^	14.30 ± 0.59^d^	84.99 ± 0.36^ab^	0.69 ± 0.02^c^
		6	1.62 ± 0.07^f^	13.33 ± 0.00^e^	164.11 ± 0.90^a^	16.64 ± 0.91^c^	82.42 ± 0.80^c^	0.92 ± 0.10^bc^
		9	1.83 ± 0.00^e^	13.67 ± 0.00^c^	132.69 ± 6.36^de^	13.68 ± 0.28^d^	85.32 ± 0.31^ab^	0.99 ± 0.61^bc^
	OH	3	2.03 ± 0.01^bc^	14.17 ± 0.00^b^	126.49 ± 0.70^f^	16.94 ± 0.70^c^	84.67 ± 0.00^ab^	1.37 ± 0.55^ab^
		6	2.05 ± 0.00^ab^	14.17 ± 0.00^b^	144.80 ± 0.70^c^	17.08 ± 0.14^c^	84.55 ± 0.00^bc^	1.52 ± 0.46^a^
		9	2.00 ± 0.00^cd^	14.17 ± 0.00^b^	156.37 ± 3.36^b^	13.70 ± 0.49^d^	86.18 ± 0.70^a^	1.02 ± 0.77^bc^
	OHP	3	2.10 ± 0.00^ab^	13.25 ± 0.00^e^	132.19 ± 0.00^de^	19.12 ± 0.36^ab^	78.69 ± 0.55^d^	1.53 ± 0.00^a^
		6	2.01 ± 0.28^cd^	13.55 ± 0.16^d^	130.75 ± 1.07^ef^	17.59 ± 2.59^bc^	79.89 ± 2.58^d^	0.98 ± 0.00^bc^
		9	1.95 ± 0.00^d^	14.17 ± 0.00^b^	136.97 ± 0.00^d^	20.53 ± 0.63^a^	76.51 ± 1.20^e^	1.25 ± 0.07^ab^
30	Control	0	0.55 ± 0.00^a^	16.83 ± 0.00^bc^	78.64 ± 0.00^e^	2.31 ± 0.00^f^	96.93 ± 0.07^a^	2.38 ± 0.00^a^
	OP	3	0.43 ± 0.01^bcd^	16.65 ± 0.24^bc^	100.00 ± 0.00^a^	2.91 ± 0.01^e^	94.84 ± 0.73^cd^	1.36 ± 0.56^c^
		6	0.42 ± 0.01^bcd^	17.16 ± 0.36^bc^	86.97 ± 0.00^c^	3.69 ± 0.06^b^	95.57 ± 0.00^b^	2.14 ± 0.00^ab^
		9	0.28 ± 0.00^e^	17.37 ± 0.00^b^	86.97 ± 0.00^c^	2.96 ± 0.00^e^	94.48 ± 0.00^d^	1.53 ± 0.00^bc^
	OH	3	0.41 ± 0.12^cd^	16.66 ± 0.51^bc^	84.43 ± 1.71^d^	3.30 ± 0.00^c^	95.47 ± 0.45^b^	1.47 ± 0.00^bc^
		6	0.51 ± 0.00^ab^	16.40 ± 0.87^c^	92.48 ± 1.41^b^	3.72 ± 0.00^b^	93.81 ± 0.34^e^	2.59 ± 0.00^a^
		9	0.37 ± 0.00^d^	17.02 ± 0.00^bc^	100.00 ± 0.00^a^	3.04 ± 0.00^d^	95.11 ± 0.07^bc^	2.04 ± 0.00^abc^
	OHP	3	0.40 ± 0.01^d^	18.95 ± 0.00^a^	100.00 ± 0.00^a^	3.85 ± 0.00^a^	93.55 ± 0.00^e^	2.28 ± 0.08^a^
		6	0.49 ± 0.00^abc^	14.17 ± 0.00^d^	100.00 ± 0.00^a^	3.68 ± 0.07^b^	95.53 ± 0.00^b^	2.15 ± 0.07^ab^
		9	0.43 ± 0.00^bcd^	17.02 ± 0.00^abc^	86.97 ± 0.00^c^	3.30 ± 0.00^c^	95.12 ± 0.00^bc^	2.53 ± 0.56^a^

OP, dough with short-chain inulin; OH, dough with natural inulin; OHP, dough with long-chain inulin. Each value is expressed as the mean value ± standard deviation. Mean values with different lower case letters within a column indicate a significant (*P* < 0.05) difference.

Here, the proportions of A_21_ and A_23_ in the control consistently decreased, while A_22_ demonstrated an increasing tendency with the duration of freezing time. These findings suggest a shift of bound water to semi-bound water and free water within the dough matrix during freezing. This shift indicates a reduced capacity for water retention and an increase in water loss within the dough over time. Adding inulin led to an increase in A_21_ values and a decrease in A_22_ and A_23_ values of the dough after 30 days of freezing. This is due to the hydrogen bonding between inulin and water (Luo et al., 2017). From 0 to 30 days of freezing, the control group displayed significant reductions in A_21_ by 87.62% and A_23_ by 17.37%, while A_22_ increased by 25.10%. In contrast, the dough containing 6% OP, 6% OH, and 3% OHP displayed the least trend of water change. Specifically, its A_21_ decreased by 82.97%, 80.97%, and 81.16%, respectively. A_22_ increased by 16.86%, 13.31%, and 16.38%, while A_23_ decreased by 4.62%, 5.98%, and 8.81%, respectively. This suggests that inulin retards the water change in frozen dough.

Overall, inulin delays the fluidity of the water in the frozen dough. Specifically, with the addition of 6% OP, 6% OH, and 3% OHP, the water of the dough binds more tightly to the other components. These combined effects effectively delay water migration, safeguard the structure of the gluten network, and bolster the stability of the frozen dough (Lou et al., 2023).

### 3.5 Microstructure of dough

**Fig. 3:**
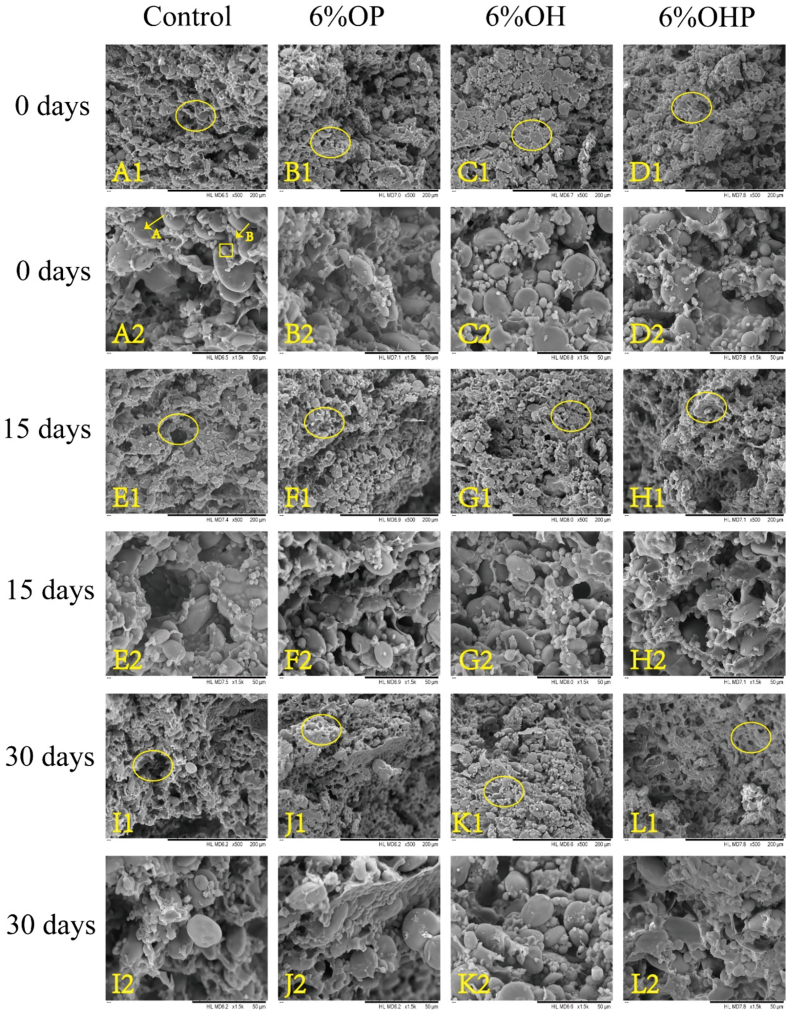
**SEM micrographs of dough containing 6% OP, 6% OH, and 3% OHP after freezing for 0 days (A-D), 15 days (*E*-H), and 30 days (I-L).** A, E, and I, the control dough; B, F, and J, 6% OP dough; C, G, and K, 6% OH dough; D, H, and L, 3% OHP dough. OP, dough with short-chain inulin; OH, dough with natural inulin; OHP, dough with long-chain inulin.

The microstructure of dough containing 0%, 6% OP, 6% OH, and 3% OHP after being frozen for 0, 15, and 30 days is shown in Fig. 3. The control dough displayed a complete gluten network structure, with evenly distributed larger type A wheat starch grains and smaller type B wheat starch grains, forming complete and cohesive starch particles within the dough (Fig. 3A). After the incorporation of inulin, the gluten network became more continuous (Figs. 3B, C, and D). This is due to the hydrogen bonding and hydrophobic interactions between OH and proteins, which promote a tighter network structure. During cryopreservation, the microstructure of the control was mechanically damaged due to the ice crystals, and the starch particles and gluten structures were separated (Fig. 3E). After 30 days of freezing, the control group's gluten network displayed cracks and significant depressions in its microstructure (Fig. 3I). This degradation results from disulfide bond breakage, gluten depolymerization, varying degrees of starch particle exposure, and the formation of ice crystals, ultimately leading to the disruption of the gluten network. In contrast, the gluten network within the inulin-enriched dough remained largely intact, particularly with OH (Fig. 3L). Inulin alters the distribution of water in the dough, resulting in free water predominantly existing in the form of small ice crystals within the gluten network structure. This adjustment significantly contributes to preserving a robust gluten network structure during freezing.

The results demonstrates that the addition of inulin has an effect on the microstructure of the frozen dough, leading to a more uniform distribution of starch particles as well as an enhanced gluten network. This finely uniform arrangement within the tissue holds the potential to enhance both the texture and taste of the final product. These findings align with the outcomes of the farinograph and extensograph properties of dough, suggesting that inulin can serve as an enhancer, effectively improving the quality of frozen dough and its resulting products. Particularly noteworthy was the optimal effect observed with 6% OH among the tested variants.

### 3.6 Colorimetry of steamed bread

The color of the steamed bread crust plays a pivotal role in determining its quality and consumer acceptance (Hsu, Chang, & Shiau, 2019). The color of the steamed bread crust containing inulin is given in Table S2. Adding inulin significantly (*P* < 0.05) increased the L* values of unfrozen steamed bread compared with that of the control, indicating that inulin positively affects the brightness of the steamed bread crust color. As the freezing time increased, the brightness value of the steamed bread decreased. This phenomenon could be attributed to the formation of ice crystals within the steamed bread during freezing. Subsequent recrystallization of these ice crystals leads to the formation of larger crystals, causing mechanical damage to the dough. As a result, the crust of the steamed bread undergoes shrinkage and collapse, ultimately deteriorating its color (Zhao et al., 2022). After being frozen from 0 to 15 days, the L* values of control decreased by 12.43%, while the L* value of 6% OP, 6% OH, and 3% OHP showed relatively smaller decreases of 5.37%, 6.96%, and 5.50%, respectively. These findings suggest that inulin may inhibit water crystallization and maintain the quality of steamed bread by mitigating the decrease in brightness during freezing.

Here, at the same freezing time, the a* value of the steamed bread containing inulin exhibited a notably higher a* value compared with that of the control. Moreover, as the substitution level of inulin increased, there was a discernible upward trend in the a* value of the steamed bread. This is consistent with the observations reported by Luo, Liang, Xu, Kou, et al. (2017). The ΔE*_ab_ value of 6% OH steamed bread changed non-obviously compared with that of the control at the same freezing time. This implies that OH don't significantly impact the color of the steamed bread crust during freezing. Furthermore, the ΔE*_ab_ values of steamed bread containing OHP were notably higher than those of steamed bread with OP and OH additions. These findings suggest that adding OHP disrupts the formation of the gluten network, resulting in a more noticeable change in color perception.

### 3.7 Specific volume and spread ratio of steamed bread

**Fig. 4:**
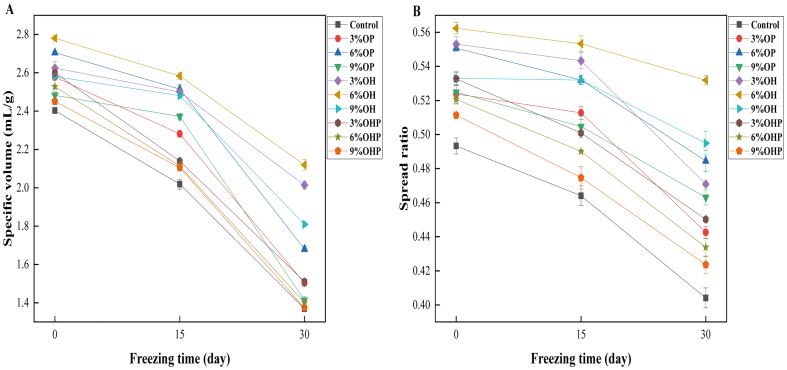
**Specific volume and spread ratio of steamed bread made from dough with inulin at different freezing time.** OP, steamed bread made from dough with short-chain inulin; OH, steamed bread made from dough with natural inulin; OHP, steamed bread made from dough with long-chain inulin.

The specific volume and the spread ratio of steamed bread are indicators of the expansion degree and retention ability of dough volume, which directly correlate with the external texture, internal structure, and taste profile of steamed bread (Sun et al., 2020). The specific volume and spread ratio of steamed bread made from dough containing inulin at different freezing time are shown in Fig. 4. The specific volume and spread ratio of steamed bread significantly increased with the increase in inulin substitution level (Fig. 4). On day 0, the specific volume of steamed bread added with 6% OP, 6% OH, and 3% OHP was the highest, with increases of 12.91%, 16.25%, and 8.33%, respectively, compared with the control (Fig. 4A). Concurrently, these inulin-enriched steamed bread also displayed the most significant spread ratio (Fig. 4B), escalating by 12.24%, 14.29%, and 8.16%, respectively, compared with the control. The reason may be that the supplementation of inulin strengthens the network structure of the dough, enabling increased retention of CO_2_ and resulting in well-shaped steamed bread. Furthermore, inulin's role in reducing water absorption within the dough promotes the development of a robust network structure (Peressini & Sensidoni, 2009). At the same freezing time, the specific volume of steamed bread initially increased and then decreased with the increase in OP and OH substitution levels. However, when the substitution level of OHP exceeded 3%, the specific volume of steamed bread gradually diminished. This reduction in specific volume leads to a denser core and reduced gas chamber volume, resulting in increased hardness, which is consistent with the texture results.

Here, as frozen storage time increased, both the specific volume and spread ratio of the control exhibited a declining trend. This decline stemmed from reduced yeast activity, the disruption of the dough's three-dimensional structure, and damage to the gluten network during frozen storage. From 0 to 30 days of freezing, the specific volume of the control decreased by 42.91%. However, steamed bread added with 6% OP, 6% OH, and 3% OHP decreased by 37.64%, 23.66%, and 41.54%, respectively. This suggests that inulin could mitigate the damage to steamed bread caused by freezing.

In summary, adding inulin increases the specific volume of the steamed bread made from frozen dough. Notably, incorporating 6% OP, 6% OH, and 3% OHP displays the most significant improvements in the quality of the steamed bread.

### 3.8 Texture of steamed bread

**Fig. 5:**
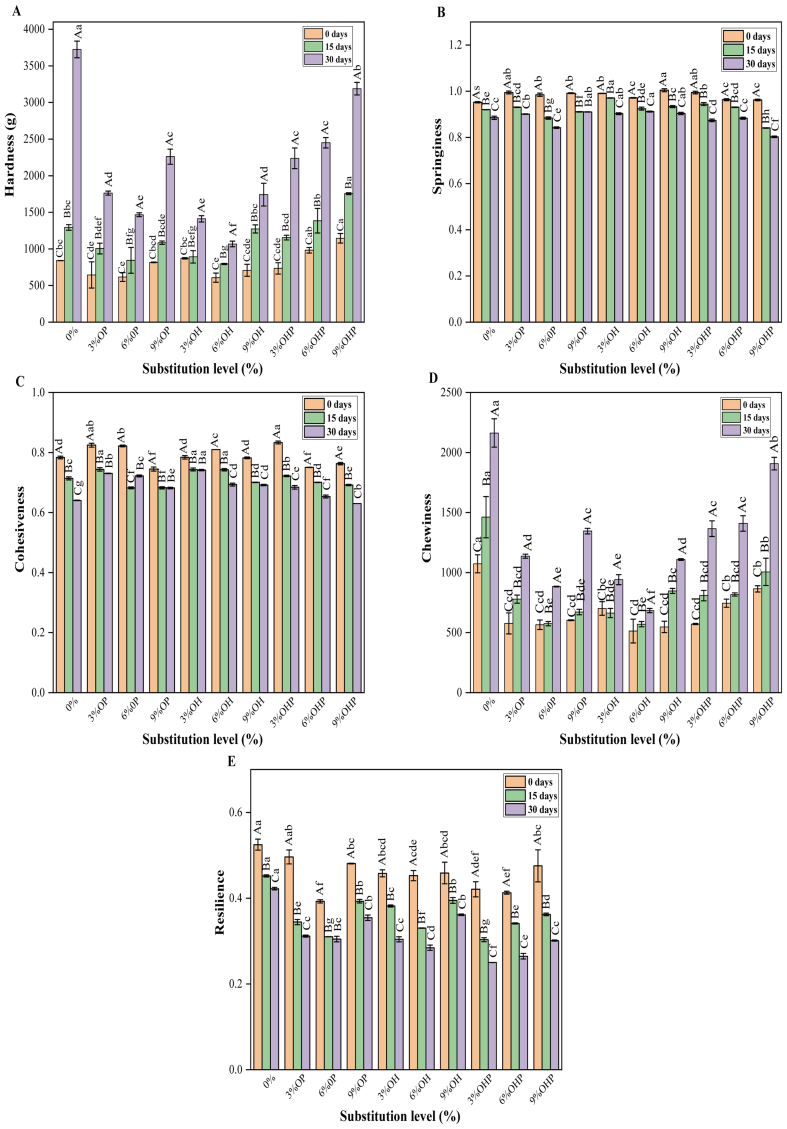
**Textural characteristics of steamed bread made from dough with inulin at different freezing time.** OP, steamed bread made from dough with short-chain inulin; OH, steamed bread made from dough with natural inulin; OHP, steamed bread made from dough with long-chain inulin. Different upper case letters indicate significant (*P* < 0.05) differences at different freezing time for the same DP of inulin. Different lower case letters indicate significant (*P* < 0.05) differences between different DP of inulin at same freezing time.

Texture profile analysis is defined as the combination of sensory perception with its mechanical and geometric characteristics, serving as a crucial index for evaluating the properties of steamed bread (Chen et al., 2020). The influence of inulin with different DP and different substitution levels on the textural characteristics of steamed bread is presented in Fig. 5. The addition of inulin significantly (*P* < 0.05) changed the hardness, chewiness, and resilience of steamed bread. At the same freezing time, the hardness of steamed bread first decreased and then increased with the increase of OP and OH substitution levels, reaching its lowest at 6% substitution. This is because of the water-retaining properties of inulin (Kou et al., 2019). On day 0, the hardness of the steamed bread was significantly (*P* < 0.05) higher than that of the control when the OHP substitution level was ≥3%. However, after 30 days of freezing, the hardness of the steamed bread containing OHP was lower than that of the control. This finding indicates that OHP additionally demonstrates a safeguarding impact on the frozen dough-derived steamed bread. The chewiness of steamed bread added with OHP was higher than that of steamed bread with OP and OH (Fig. 5C). This can be attributed to the higher DP and superior gelling properties of OHP. In one study, inulin gel with a higher molecular weight had greater hardness and chewiness (Mensink et al., 2015).

Here, from 0 to 30 days of freezing, the hardness of the control increased by 343.24%. By contrast, the hardness of steamed bread supplemented with 6% OP, 6% OH, and 3% OHP increased by 138.07%, 75.73%, and 204.78%, respectively. This demonstrates that incorporating OP, OH, and OHP mitigate the increase in the hardness of steamed bread, with OH having the most notable effect. There are many factors affecting the texture of steamed bread, such as water migration, water sublimation, amylose recrystallization, ice crystal growth, and interaction of gluten and starch. The addition of inulin inhibits the formation of ice crystals and slows the flow of water in the dough, thereby reducing the degree of damage caused by freezing to the gluten network, and ultimately leading to a reduction in the hardness of the steamed bread.

Resilience reflects the ability of steamed bread to quickly recover its deformation after compression. After 30 days of freezing, the resilience of steamed bread containing inulin was significantly (*P* < 0.05) reduced compared with the control (Fig. 5E). Steamed bread with a lower recovery rate was more prone to damage. Additionally, from 0 to 30 days of freezing, the springiness and cohesiveness of steamed bread containing inulin remained unchanged compared with those of the control. This suggests that the springiness of the steamed bread is not compromised by the addition of inulin.

In summary, when considering the texture attributes of steamed bread alongside the rheological properties of dough, inulin emerges as a multifaceted ingredient. It not only impedes ice crystal formation and starch recovery but also fortifies the gluten network, thereby enhancing the structural integrity of the steamed bread. As evidenced by the texture, 6% OP, 6% OH, and 3% OHP demonstrates the most notable enhancement effects.

### 3.9 Mechanism

Based on these results, the potential mechanisms by which inulin improves the rheological properties and water distribution of frozen dough are as follows. The polar groups, such as hydroxyl groups, in the polysaccharide chain structure of inulin enhance its interaction with water molecules, improving its hydrophilicity. This characteristic allows inulin to compete with proteins and starches in wheat flour for binding with water molecules, thereby decreasing the water absorption rate of the dough and affecting its farinograph characteristics.

Hydrophilic groups in inulin interact with water, starch, and protein to form complexes, optimizing the hydration state of the gluten network structure in frozen dough and altering its rheological characteristics. OHP has a higher number of fructose units and longer chains compared with OP and OH, resulting in stronger interactions with large molecules such as proteins and sugars, consequently exerting a greater influence on the rheological characteristics of the dough, particularly on T_23_. Inulin combines hydrogen bonds and hydrophobicity to form a denser network structure, enhancing water molecule retention and delaying changes in water distribution in frozen dough. The water bound by inulin reduces its freezability, leading to the formation of smaller ice crystals during frozen storage and releasing less water upon thawing. Consequently, this process enhances the gluten network structure. Furthermore, inulin enhances the retention of carbon dioxide in steamed bread, resulting in bread with a higher specific volume and lower hardness.

## 4 Conclusions

In this study, we examined the effects of OP, OH, and OHP, as well as freezing time, on the farinograph and extensograph characteristics of flour. Additionally, we investigated the impacts of inulin on the rheological properties, water distribution, and microstructure of frozen dough, as well as the quality of the final steamed bread. Consequently, we explored the mechanisms underlying the effects of inulin and freezing on dough characteristics and the quality of steamed bread. The results revealed significant alterations in farinograph and extensograph properties, rheological characteristics, water distribution within the frozen dough, and the overall quality of steamed bread when substituting wheat flour with inulin of varying DP. Notably, the water absorption of the dough consistently decreased with higher levels of the three inulin substitutions. Adding OP, OH, and OHP resulted in an increase in the DT, ST, and FQN of the dough while reducing its SD. After a fermentation time of 135 min, the dough enriched with inulin exhibited lower E but showed higher R, R/E, and extension energy compared with those of the control. Furthermore, the G' and G" of the control consistently decreased, reaching a minimum after 30 days of freezing. In contrast, the decline in G' and G" values of the inulin-enriched dough was comparatively restrained. Frozen dough supplemented with 6% OP, 6% OH, and 3% OHP experienced minimal water fluctuation, effectively preserving the integrity of the gluten network.

SEM analysis revealed that frozen dough fortified with inulin exhibited resilience against ice crystal damage, displayed improved continuity, and maintained an intact gluten network, with the effect being particularly noticeable with 6% OH. Additionally, both OP and OH, as well as OHP, collectively contributed to enhancing the L* value of the steamed bread. Interestingly, OHP exhibited a higher ΔE*_ab_ compared with OP and OH, indicating that OP and OH have a more pronounced impact on the color of the steamed bread than OHP. Furthermore, the specific volume and spread ratio of the inulin-enriched steamed bread consistently exceeded those of the control. The texture profile analysis results highlighted that after 30 days of freezing, all three inulin types contributed to reducing the hardness of the steamed bread. Among them, 6% OP and OH exhibited optimal substitution levels, whereas 3% OHP emerged as the most favorable substitution level. These findings provide valuable insights into the potential application of OP, OH, and OHP in frozen flour products. Future research should explore the effects of inulin on the storage characteristics and functional properties of steamed bread.

## CRediT authorship contribution statement

**Qing Yang:** Writing – original draft, Validation, Resources, Methodology, Formal analysis, Data curation, Conceptualization. **Jinying Guo:** Writing – review & editing, Supervision, Project administration, Investigation, Funding acquisition, Conceptualization. **Fan Zhang:** Visualization, Validation, Software, Methodology, Data curation, Conceptualization. **Fen Zhao:** Software, Resources, Methodology, Data curation, Conceptualization. **Gege Zhang:** Supervision, Software, Resources, Methodology, Formal analysis, Conceptualization.

## Declaration of competing interest

The authors declare that they have no known competing financial interests or personal relationships that could have appeared to influence the work reported in this paper.

## Data Availability

Data will be made available on request.

## Appendix A Supplementary data

Supplementary material Image 1
