# Supplementary material for: Inulin with different degrees of polymerization as a functional ingredient: Evaluation of flour, dough, and steamed bread characteristics during freezing
Source: Food Chem X. 2024 May 3;22:101431. doi: 10.1016/j.fochx.2024.101431 (PMC11101675; doi:10.1016/j.fochx.2024.101431)
Supplement: Supplementary file 1 — Supplementary material [file mmc1.docx]

**Table S1 Farinographic parameters of dough containing inulin.**

| Inulin | Substitution level (%) | WA (%) | DT (min) | ST (min) | SD (FU) | FQN (min) |
| --- | --- | --- | --- | --- | --- | --- |
| Control | 0 | 60.47 ± 1.61^a^ | 6.7 ± 0.00^f^ | 9.4 ± 0.21g | 65 ± 3.54^a^ | 116 ± 3.54^e^ |
|  |  |  |  |  |  |  |
| OP | 3 | 57.57 ± 0.14^b^ | 7.9 ± 0.07^d^ | 12.8 ± 0.35^cd^ | 53 ± 0.71^bc^ | 148 ± 1.41^c^ |
|  | 6 | 53.30 ± 0.71^c^ | 8.1 ± 0.07^cd^ | 12.2 ± 0.21^de^ | 43 ± 2.21^de^ | 138 ± 2.12^d^ |
|  | 9 | 50.40 ± 0.14^d^ | 8.0 ± 0.00^cd^ | 9.9 ± 0.35^fg^ | 55 ± 1.41^b^ | 137 ± 1.41^d^ |
|  |  |  |  |  |  |  |
| OH | 3 | 57.63 ± 0.71^b^ | 7.8 ± 0.14^d^ | 14.0 ± 0.21^b^ | 46 ± 1.41^de^ | 172 ± 2.12^b^ |
|  | 6 | 54.34 ± 0.33^c^ | 7.4 ± 0.14^e^ | 11.8 ± 0.49^e^ | 54 ± 3.54^bc^ | 137 ± 4.95^d^ |
|  | 9 | 48.17 ± 0.23^e^ | 8.9 ± 0.21^a^ | 17.3 ± 0.49^a^ | 30 ± 4.95^f^ | 225 ± 6.36^a^ |
|  |  |  |  |  |  |  |
| OHP | 3 | 57.52 ± 0.45^b^ | 8.3 ± 0.07^bc^ | 13.2 ± 0.21^c^ | 42 ± 0.71^e^ | 165 ± 2.83^b^ |
|  | 6 | 54.32 ± 0.59^c^ | 8.5 ± 0.14^b^ | 11.8 ± 0.14^e^ | 54 ± 2.12^bc^ | 140 ± 0.00^d^ |
|  | 9 | 51.35 ± 0.21^d^ | 8.2 ± 0.14^c^ | 10.5 ± 0.35f | 48 ± 1.41^cd^ | 135 ± 1.41^d^ |

OP, dough with short-chain inulin; OH, dough with natural inulin; OHP, dough with long-chain inulin. WA, water absorption; DT, development time; ST, stable time; SD, softening degree; FQN, farinograph quality number. The values represent the mean values ± standard deviation (n = 3). Mean values with different lower case letters within a column indicate a significant (*P* < 0.05) difference.

**Table S2 Colorimetry of steamed bread crust steamed bread made from dough with inulin at different freezing time.**

| Freezing time (d) | Inulin | Substitution level (%) | Crust color | | | |
| --- | --- | --- | --- | --- | --- | --- |
|  |  |  | L^*^ | a^*^ | b^*^ | △E^*^_ab_ |
| 0 | Control | 0 | 71.36 ± 3.73^d^ | -1.36 ± 0.14^d^ | 12.08 ± 1.28^a^ | - |
|  | OP | 3 | 72.17 ± 1.68^d^ | -1.13 ± 0.17^abc^ | 9.84 ± 1.59^cd^ | 4.09 ± 2.43^cde^ |
|  |  | 6 | 76.06 ± 1.05^bc^ | -1.09 ± 0.05^ab^ | 11.74 ± 0.84^ab^ | 5.11 ± 2.89^cd^ |
|  |  | 9 | 76.27 ± 1.05^bc^ | -0.99 ± 0.04^a^ | 11.14 ± 0.55^abc^ | 5.31 ± 3.69^cd^ |
|  | OH | 3 | 76.48 ± 0.75^abc^ | 1.04 ± 0.01^ab^ | 11.01 ± 0.28^abcd^ | 3.85 ± 0.66^cde^ |
|  |  | 6 | 75.21 ± 0.71^c^ | 1.20 ± 0.13^bcd^ | 9.36 ± 1.20^d^ | 3.30 ± 0.49^de^ |
|  |  | 9 | 79.17 ± 0.75^a^ | 1.14 ± 0.02^abc^ | 9.66 ± 0.73^cd^ | 6.68 ± 0.43^bcd^ |
|  | OHP | 3 | 76.48 ± 1.19^abc^ | -1.28 ± 0.10^cd^ | 9.99 ± 0.32^cd^ | 8.20 ± 0.28^abc^ |
|  |  | 6 | 78.68 ± 0.92^ab^ | 1.15 ± 0.09^abc^ | 10.40 ± 0.60^bcd^ | 12.09 ± 0.13^a^ |
|  |  | 9 | 77.37 ± 1.42^abc^ | 1.20 ± 0.13^bcd^ | 10.41 ± 0.78^bcd^ | 10.33 ± 0.46^ab^ |
| 15 | Control | 0 | 62.49 ± 2.53^d^ | -1.57 ± 0.29^ab^ | 11.91 ± 1.80^ab^ | 3.84 ± 0.06^c^ |
|  | OP | 3 | 67.86 ± 0.25^c^ | -1.39 ± 0.06^a^ | 9.47 ± 4.02^b^ | 4.52 ± 2.24^bc^ |
|  |  | 6 | 71.97 ± 1.65^ab^ | -1.33 ± 0.17^a^ | 11.79± 3.70^ab^ | 4.68 ± 2.60^bc^ |
|  |  | 9 | 68.49 ± 2.27^c^ | -1.31 ± 0.08^a^ | 13.30 ± 0.84^ab^ | 4.59 ± 0.94^bc^ |
|  | OH | 3 | 70.42 ± 2.04^abc^ | -1.56 ± 0.10^ab^ | 12.84 ± 1.63^ab^ | 3.24 ± 1.67^c^ |
|  |  | 6 | 69.97 ± 0.83^bc^ | -1.65 ± 0.09^b^ | 12.35 ± 0.60^ab^ | 2.97 ± 0.33^c^ |
|  |  | 9 | 70.34 ± 0.83^abc^ | -1.47 ± 0.07^ab^ | 12.85 ± 2.21^ab^ | 3.84 ± 0.06^c^ |
|  | OHP | 3 | 72.27 ± 2.06^ab^ | -1.47 ± 0.07^ab^ | 12.17 ± 0.52^ab^ | 8.15 ± 1.93^a^ |
|  |  | 6 | 73.30 ± 1.37^a^ | -1.49 ± 0.08^ab^ | 13.87 ± 1.86^a^ | 7.78 ± 0.16^ab^ |
|  |  | 9 | 69.29 ± 0.52^bc^ | -1.46 ± 0.06^ab^ | 13.31 ± 1.07^ab^ | 6.00 ± 1.53^bc^ |
| 30 | Control | 0 | 70.81 ± 0.93^c^ | -1.12 ± 0.07^e^ | 13.80 ± 0.60^a^ | 6.46 ± 0.78^a^ |
|  | OP | 3 | 72.94 ± 0.49^bc^ | -1.04 ± 0.07^de^ | 11.95 ± 1.38^ab^ | 4.27 ± 0.91^abc^ |
|  |  | 6 | 73.25 ± 2.47^bc^ | -0.65 ± 0.04^a^ | 13.43 ± 1.53^a^ | 3.00 ± 1.65^bc^ |
|  |  | 9 | 74.11 ± 1.45^b^ | -0.75 ± 0.26^ab^ | 11.64 ± 0.82^ab^ | 3.77 ± 2.14^abc^ |
|  | OH | 3 | 72.00 ± 2.46^bc^ | 0.87 ± 0.02^bcd^ | 13.08 ± 0.70^ab^ | 2.53 ± 1.27^bc^ |
|  |  | 6 | 72.56 ± 2.43^bc^ | 0.80 ± 0.04^abc^ | 12.23 ± 3.19^ab^ | 1.73 ± 0.91^c^ |
|  |  | 9 | 73.22 ± 1.61^bc^ | 0.97 ± 0.06^cde^ | 9.87 ± 1.85^b^ | 2.26 ± 0.52^c^ |
|  | OHP | 3 | 73.23 ± 1.60^bc^ | 0.88 ± 0.05^bcd^ | 11.28 ± 0.69^ab^ | 4.05 ± 2.03^abc^ |
|  |  | 6 | 74.85 ± 0.66^ab^ | 0.92 ± 0.04^bcd^ | 11.84 ± 2.83^ab^ | 4.31 ± 0.77^abc^ |
|  |  | 9 | 77.43 ± 1.03^a^ | 0.80 ± 0.03^abc^ | 13.80 ± 0.60^a^ | 5.31 ± 0.46^ab^ |

OP, steamed bread made from dough with short-chain inulin; OH, steamed bread made from dough with natural inulin; OHP, steamed bread made from dough with long-chain inulin. Each value is expressed as the mean value ± standard deviation (n = 3). Mean values with different lower case letters within a column indicate a significant (*P* < 0.05) difference.
